# Selection Criteria for the Radical Treatment of Locally Advanced Rectal Cancer

**DOI:** 10.1155/2011/678506

**Published:** 2011-10-13

**Authors:** Mansel Leigh Davies, Dean Harris, Mark Davies, Malcolm Lucas, Peter Drew, John Beynon

**Affiliations:** ^1^Department of Colorectal Surgery, Singleton Hospital, Swansea SA2 8QA, UK; ^2^Department of Urology, Morriston Hospital, Swansea SA6 6NL, UK; ^3^Department of Plastic Surgery, Morriston Hospital, Swansea SA6 6NL, UK

## Abstract

There are over 14,000 newly diagnosed rectal cancers per year in the United Kingdom of which between 50 and 64 percent are locally advanced (T_3_/T_4_) at presentation. Pelvic exenterative surgery was first described by Brunschwig in 1948 for advanced cervical cancer, but early series reported high morbidity and mortality. This approach was later applied to advanced primary rectal carcinomas with contemporary series reporting 5-year survival rates between 32 and 66 percent and to recurrent rectal carcinoma with survival rates of 22–42%. The Swansea Pelvic Oncology Group was established in 1999 and is involved in the assessment and management of advanced pelvic malignancies referred both regionally and UK wide. This paper will set out the selection, assessment, preparation, surgery, and outcomes from pelvic exenterative surgery for locally advanced primary rectal carcinomas.

## 1. Introduction

There are over 14,000 newly diagnosed rectal cancers per year in the United Kingdom [1] of which between 50 and 64 percent are locally advanced (T_3_/T_4_) at presentation [2, 3]. Preoperative imaging now accurately identifies involvement of adjacent pelvic organs (T_4_ disease) which precludes conventional resection strategies. Local recurrence after previous rectal cancer surgery, with an incidence of 5–15 percent, shares a common approach to the evaluation and surgical approach to primary T4 rectal cancer.

The state-of-the-art approach to rectal cancer includes high quality audited total mesorectal excision (TME) surgery, cylindrical abdominoperineal excision, and extended resections, which are beyond normal anatomical boundaries. The primary aim of surgery is to obtain clear resection margins to avoid future local recurrence. Pelvic exenterative surgery was first described by Brunschwig in 1948 [4] for advanced cervical cancer, but early series reported high morbidity and mortality. This approach was later applied to advanced primary rectal carcinomas with contemporary series reporting 5-year survival rates between 32 and 66 percent [5, 6] and to recurrent rectal carcinoma with survival rates of 22–42% [7]. 

## 2. Aim

The aim of this paper is to set out the criteria for selection of patients suitable for radical treatment of primary locally advanced rectal carcinoma to offer the possibility of cure. It will also set out to establish the need for this surgery to be undertaken in high-volume centres with a specialist interest in the management of advanced pelvic malignancy (including urogenital malignancies), the need for a specialist pelvic oncology MDT, and the need for a team with an expressed interest in these challenging cases.

## 3. Approach

The Calman-Hine Framework [8] has recommended that cancer services be delivered via a site-specific multidisciplinary team (MDT). Others have suggested however that, in the case of advanced pelvic malignancy, a dedicated multispeciality team would be more appropriate for the assessment and management of these difficult cases [9, 10]. To this end the Swansea Pelvic Oncology Group was established in 1999 and is involved in the assessment and management of advanced pelvic malignancies referred both regionally and UK wide.

With small numbers of patients suitable for exenterative surgery, the role of a multispecialist team covering a large geographical area cannot be underestimated. This approach leads to the development of substantial experience and delivers high-volume and high-quality surgery [11, 12]. The group has operated on over 350 pelvic oncology patients since 1999, averaging 32 cases per year. The involved specialities in this group include colorectal, gynae-oncology, urological, plastic reconstructive, orthopaedic, and neurosurgical surgical teams together with radiology, pathology, and oncology support. The team should meet on a weekly basis in an MDT forum to discuss optimal treatment of these highly individualised patients.

## 4. Extent and Justification for Exenterative Surgery

It is estimated that approximately 10% of all colorectal carcinomas are found to be locally advanced and invading adjacent structures but without metastatic disease at presentation [13]. It has long been known that there are variants of colorectal cancer that favour local invasion without metastatic potential making this group particularly suited to local radical surgery which translates into long-term survival [14]. 

Although many tumours exhibit adhesions to adjacent structures, it would not be true to say that all of these are malignant invasion. The incidence of these adhesions being malignant varies in different series between 40 and 84% [13]. However, in these cases it is certainly true that less than radical en bloc resection increases the local recurrence rate and compromises patient survival [15–17]. In one study 5-year survival in en bloc resection was in the order of 61%, but this fell to 23% when tumours were separated from the organs to which they were adherent, similarly with local recurrence, en bloc resection had a local recurrence rate of 36% versus 77% if tumours were separated [15]. 

Furthermore, the evidence suggests that before setting out on pelvic exenterative surgery the intent at surgery must be to undertake a curative resection with histologically clear resection margins. This needs to be predicted preoperatively in order to keep those patients having a trial dissection or “open and close” surgery to a minimum. Resection planes can be accurately predicted with modern high-resolution imaging. Results from the Swansea Pelvic Oncology Group [11] demonstrate that resections for advanced pelvic malignancy with clear margins demonstrate a 5-year survival of 53%. There was no survival advantage in patients with positive margins, palliative surgery, and no surgery with median survivals of 12 months, 9 months, and 13 months, respectively. Negative resection margins and node-negative status were associated with a 53% five-year survival, reflecting acceptable long-term survival in a group whose only other option is palliation.

## 5. Assessment and Imaging

Locally advanced rectal cancer may involve such adjacent organs as the bladder, prostate, seminal vesicles, urethra, ureter, uterus and adnexa and sacrum. Direct invasion of these structures may be amenable to radical exenterative surgery and potential cure in appropriately selected patients. Each case of locally advanced rectal carcinoma referred for consideration for radical surgical treatment should be assessed on an individual basis and independently by the multispecialty team. The primary function of this assessment is to review the clinical, pathological and radiological findings of the patients' investigations in order to make a judgement of the patients' suitability to undergo a potentially curative pelvic resection. Patient fitness for major exenterative surgery is of paramount concern for optimal results. We and other centres have begun utilising cardiopulmonary exercise (CPEX) testing in this regard [18].

Assessment of suitability for multivisceral resectional surgery takes the form of a multimodality assessment involving clinical evaluation (including digital rectal examination, examination under anaesthetic of the rectum and vagina), endoscopic assessment, radiological assessment (high-resolution MRI/endorectal ultrasound for local assessment and computed tomography (chest/abdo/pelvis)/PET CT for assessment of distant disease) and pathological assessment. The primary aim of this assessment is to select out those cases in which a curative resection is not feasible in order to avoid unnecessary open and close type surgical interventions and avoid exposing the patient to the risk of surgical morbidity and mortality in cases in which surgery would be ultimately futile.

## 6. Clinical Assessment

Clinical assessment allows the experienced clinician to correlate the findings of other investigative modalities with their clinical findings and allows a better understanding of the tumours relationship with the structures surrounding it. It allows for a subjective assessment of fixation which only clinical examination is able to achieve as other investigations are only able to suggest the possibility of tumour fixation. Fixation in itself is suggestive that a lesion may be less likely to be amenable to curative resection but cannot on its own contraindicate the possibility of surgical intervention. Clinical assessment of fixation can be graded in accordance with the criteria established by Nicholls et al. in that the tumour is either clinically mobile, tethered, or fixed [19]. In addition clinical examination gives the clinician an assessment of the position of the tumour to aide in clinical decision making and gives an assessment of the height of the primary tumour useful in making decisions regarding the possibility of a restorative versus nonrestorative procedure.

## 7. Endoscopic Assessment

Endoscopic assessment is useful in the assessment of tumours which may be out of the physical reach of a clinically educated finger and allows assessment of the size of tumour, the degree of luminal obstruction (which may be important if preoperative neoadjuvant therapy is required as a defunctioning stoma may be required prior to therapy commencement) and allows further pathological sampling if required. Biopsy is essential to exclude squamous cell carcinoma in anal canal lesions. In recurrent rectal cancer biopsy is not always necessary if there is enough supportive evidence such as a rising carcinoembryonic antigen (CEA) level or a positive PET-CT scan.

## 8. Local Staging

Local staging is conducted via means of a high-definition Magnetic Resonance Imaging (MRI) scan using a rectal cancer protocol. This is now well established as the standard of care as the local staging investigation of choice in rectal cancer [2, 20]. MRI is able to demonstrate the extent of local invasion into surrounding anatomical structures to a high degree of sensitivity and specificity. In addition, it is able to help predict nodal status of the tumour, assess potential for operability, exclude disease which is beyond operative salvage, and help surgical teams plan their operative approach [13]. As such it should be considered essential for assessment of locally advanced tumours both at initial staging and at subsequent restaging following neoadjuvant treatment prior to surgical intervention. High-definition MRI has been shown to have a high sensitivity in assessing the involvement of the CRM and in suggesting the involvement of adjacent organs [20, 21]. In a study of MRI involving assessment of locally advanced tumours 22 of the cohorts 28 locally advanced tumours were correctly diagnosed in advance by the use of high-definition MRI scanning. Accuracy of MRI does diminish for the lower-third rectal cancers where endorectal ultrasound (ERUS) may be superior.

In assessment of pelvic tumours for pelvic exenterative surgery, Popovich et al. [22] demonstrate an accuracy for MRI of 83%, a PPV (positive predictive value) of 56%, and an NPV (negative predictive value) of 100%. Our own data (unpublished) found that the sensitivity of MRI in this setting was 100% with a specificity of 66%. In the assessment of T staging following chemoradiotherapy, sensitivity was just 84% and specificity 63%. We are presently basing our resection strategy on the findings of the imaging and assessment at presentation rather than after neoadjuvant treatment.

Endorectal ultrasound (ERUS) has been demonstrated to accurately categorize the depth of invasion of low rectal tumours and accurately predict the involvement of adjacent structures with accuracy for T stage as high as 96% [23]. However, this does not hold true for the use of ERUS following treatment with accuracy falling as low as 50% in one study of ERUS following chemoradiotherapy in locally advanced disease [24].

## 9. Distant Staging

All patients being considered for pelvic clearance are staged with computed tomography (CT) of the chest, abdomen and pelvis to clarify local anatomy and exclude disseminated malignancy. The presence of localised metastatic disease in the liver or lung which is assessed as resectable according to current guidelines can still be considered for pelvic exenterative surgery.

Disease recurrence following exenterative surgery may result from progressive disease in occult metastases [12, 25]. As such functional imaging using fused ^18^FDG positron emission tomography CT (PET-CT) is recommended where available, in order to minimise the risk of inappropriate radical surgery on patients with distant disease which is not amenable to surgical cure [12]. This has a sensitivity of 94.6% for colorectal liver metastases [26]. In addition due to difficulties with MRI and ERUS in the reassessment of tumours treated by neoadjuvant chemoradiotherapy PET-CT may be more accurate in the local staging of tumours following neoadjuvant therapy [27, 28] with the potential to distinguish fibrosis from residual tumour. 

## 10. Classification of Exenterations

Whilst it would be advantageous to have agreed definitions of the types of exenterative surgery, this has not been possible in practice making the literature difficult to interpret. The precise pelvic compartment that the tumour is located within has a bearing on the likelihood of a complete (R0) resection and the pattern of organs requiring removal. For example, it is more challenging to obtain an R0 resection where there is sidewall involvement [29]. Most classification systems proposed relate to recurrent rectal cancer, but the principles translate to primary rectal cancer (Table 1). In the author's institution pelvic exenterations are classified into one of 5 main groups [11]: anterior pelvic exenterations (APE) which in addition to the resection of central pelvic organs includes removal of the bladder and distal ureters bilaterally; posterior pelvic exenteration (PPE) which involves removal of the central organs together with the rectosigmoid (with or without anal canal); total pelvic exenteration (TPE), a combination of both anterior and posterior pelvic exenterations; extended exenteration, which includes abdominosacral resection; individualised approaches such as rectal excision with concomitant radical prostatectomy with preservation of the bladder. 

## 11. Contraindications to Surgery

Not all patients with locally advanced or recurrent rectal cancer will be amenable to resection. Optimal outcomes result from those patients in whom clear resection margins are achieved so local staging is of paramount importance in case selection. Clearly those patients with prohibitive co-morbidities (ASA grade IV/V) will not be suitable for this arduous surgery, nor will those with advanced metastatic disease. Debulking surgery is not appropriate for patients with rectal cancer so those with extensive disease are excluded. 

Widely accepted local factors which contraindicate exenterative surgery include extension through the greater sciatic notch, encasement of external iliac vessels, para-aortic lymphadenopathy, and lower limb oedema indicating venous or lymphatic obstruction [30]. 

Whilst extensive sidewall involvement is generally considered to exclude patients for curative surgery, Solomon's unit has achieved long-term survival with no operative mortality in 36 patients treated by en bloc lateral pelvic wall dissection with internal and external iliac vascular resection [31]. 

In terms of sacral excision with invasion present above the S2-S3 junction, although this may be technically possible to resect with some centres reporting successful outcomes in small series [32], most units consider invasion above S2-S3 junction a relative contraindication to resection [30] as the morbidity of surgery outweighs the benefits.

## 12. Neoadjuvant and Adjuvant Therapy

The judicious use of radiotherapy combined with extrafascial rectal resection has been shown to optimise outcomes in rectal cancer. Preoperative radiotherapy reduces local recurrence rates [32, 33] and is considered to be more effective than postoperative irradiation [34, 35]. In the context of locally advanced rectal cancer, the decision to give preoperative radiotherapy should be based on two considerations, namely, whether the surgical resection margin is threatened and can the local recurrence rate be reduced. If circumferential margin involvement is predicted by pre-operative assessment to be absent and if involved organs can be resected en bloc, then radiotherapy may not always be necessary. 

When indicated, the author's unit offers combination therapy as external beam radiotherapy of 45 Gy in 25 fractions over 5 weeks combined with 5-FU-based chemotherapy concomitantly (either infusional 5-FU or oral capecitabine).

Following neoadjuvant therapy formal restaging should be performed routinely to reassess the resectability of the tumour. This should again be undertaken with CT, MRI, ERUS, EUA, and CT-PET as deemed appropriate by the pelvic oncology MDT.

Adjuvant therapy is indicated for involved circumferential resection margins, nodal involvement, and extramural lymphovascular invasion. Intensive follow-up should be maintained postoperatively, with clinical assessments augmented with plasma CEA estimations at 6 monthly intervals for the first 2 years and annually thereafter [12, 36]. Computed tomography follow-up scanning of the chest abdomen and pelvis should be conducted immediately following completion of adjuvant therapy and annually thereafter. Additional scanning or investigations may be undertaken during follow-up as patients symptoms dictate.

## 13. Team Approach

The development of the pelvic oncology group in Swansea has resulted in the accumulation of a large team of dedicated practitioners from various aspects of medical and surgical practice. Only with team working and major co-ordination of efforts is the practice of multivisceral resection possible for any pelvic oncologic reason including locally advanced rectal cancers. The team approach includes coordination of multiple surgical teams often from different hospitals to attend a single patient's operative journey at the right time and in the right place with the right resources. There is further team working required from professionals outside the surgical field and includes anaesthetists, intensivists, physician/gastroenterologists, haematology/blood bank, physiotherapy, stoma therapists, continence advisors, and administrative support. 

## 14. Surgery

For the radical treatment of locally advanced rectal carcinomas, there are various operations which may be deemed appropriate for each patients case based on the clinical findings and the preoperative assessments. These include total pelvic exenteration (TPE) for tumours of the rectum involving the bladder in the male or the hysterectomised female patient, posterior pelvic exenteration (PPE) for female patients in which the rectal tumour has invaded anteriorly into the uterus, cervix, or proximal vagina, or a rectal excision with en bloc prostatectomy for low rectal tumours solely invading the posterior aspect of the prostate without bladder involvement; in these cases a radical prostatectomy is undertaken with immediate bladder reconstitution. Rectal excision may take the form of either an anterior resection if the rectal tumour is high enough for safe and complete excision or as an abdominoperineal excision of the rectum (APER). Lateral pelvic lymph node dissection including obturator nodes is performed in cases where cystoprostatectomy is undertaken. 

The patient is usually positioned supine initially for both TPE and PPE with legs elevated in the Lloyd-Davies position. A midline laparotomy is performed and a full inspection of the abdominal viscera performed. Adhesions are dealt with as necessary. The sigmoid colon is mobilised initially and the ureters identified bilaterally and traced towards the pelvis. The sigmoid colon is then divided and the dissection extended into the TME plane following which dissection is tailored to each patient's individual circumstances according to the location of the primary tumour and the involved organs. In principle all involved organs should be removed en bloc without dividing the nonanatomical connections between each organ. Following pelvic dissection perineal dissection may be undertaken either in the supine Lloyd-Davies position or in the prone jack-knife position if sacrectomy is required. Low rectal anastomoses may be feasible for high rectal tumours but are avoided if there is a vesicourethral anastomosis following en bloc prostatectomy to minimise fistulation. The abdominal phase of an APER is completed with the formation of a left-sided colostomy.

## 15. Urological and Plastic Reconstruction Approaches

Urinary diversion is required in many cases, and this is performed by constructing an ileal conduit following ureteric anastomosis. Alternatively bladder augmentation may be undertaken in cases in which a large volume of bladder wall is resected without resection of the bladder in its totality. Ureteric length is another important factor to consider in reconstruction as continuity can require bladder mobilisation and reimplantation, a bladder lengthening flap such as described by Casati and Boari [37] and Ockerblad [38], or even uretero-ureteral anastomosis with a common channel to the bladder. As such a highly individualised approach is required as dictated by local circumstances.

Plastic reconstruction aims to restore anatomy and function and reduce complications. By importing well-vascularised tissue into the exenterated pelvis, dead space is minimised reducing the risk of haematoma formation and sepsis. Pelvic floor reconstruction prevents perineal herniation, while vaginal reconstruction can preserve sexual function following en bloc vaginectomy. Importing healthy tissue into an irradiated field can reduce the frequency of perineal wound complications. 

Inclusion of a reconstructive plastic surgeon in the pelvic oncology team gives the oncological surgeon freedom to excise diseased tissues as widely as is necessary to gain safe margins, without concern for how the defect will be closed. Reconstruction is best performed as part of the primary extirpative surgery, with the choice of technique depending on factors including the intended procedure, previous surgery, and comorbidities.

While most reconstructions can be planned pre-operatively, unexpected intraoperative findings necessitate a flexible approach.

Reconstructive options include omentoplasty, porcine dermal collagen implantation (Permacol, Covidien UK, Gosport, Hampshire), local advancement or rotation flaps, and distant pedicled musculocutaneous flaps. The pedicled musculocutaneous flaps commonly used include the VRAM (vertical rectus abdominus musculocutaneous) flap, the IGAM (Inferior gluteal artery musculocutaneous), or bilateral gracilis muscle flaps.

## 16. Complications and Their Management

Complications following this extensive surgery are common, and morbidity rates remain high in many series; the authors own figures demonstrate a perioperative morbidity rate of 49% but a 30-day mortality rate of 0% [12]. Others have demonstrated broadly similar morbidity rates of between 41% and 54% for overall morbidity rates and 0% to 7% for 30-day mortality rates [7, 12, 13, 40–42].

Complications of multivisceral resection include pelvic collections/abscesses without evidence of anastomotic leaks, anastomotic leaks, fistulae of both bowel and urinary tracts, necrotising fasciitis/cellulitis, pneumonia/atelectasis, bleeding, prolonged ileus, early perineal hernias, wound infection/breakdown, and flap loss [7, 12, 13, 40–42]. These early perioperative complications are managed according to standard surgical principles, but institutional experience will minimise and contain the sequalae when they occur. Sepsis from whatever cause should be drained either percutaneously or by further surgery and together with other infectious complications treated initially by broad spectrum antibiotics and subsequently by tailored antibiotic therapy according to the results of cultures. Anastomotic leaks should generally be dealt with surgically and commonly result in loss of the anastomosis and exteriorisation of the bowel. Fistulae can often be dealt with more conservatively with drainage of sepsis followed by standard fistula management culminating in delayed surgical intervention should fistula output not settle. Wound breakdown and infection can often be managed with antibiotics and topical negative pressure therapy or regular wound dressings. Flap loss may be minor, partial, or infrequently total. Often just the skin has to be sacrificed in the case of musculocutaneous reconstruction.

## 17. Quality of Life

It is striking how little attention has been made to quality of life of patients undergoing this type of surgery with its high attendant morbidity. Despite multivisceral pelvic resection being a significant surgical insult frequently resulting in postoperative morbidity and temporary and permanent stomas, there appears to be no evidence of a significant deterioration in quality of life in these patients when compared to those with rectal carcinoma which is not locally advanced and has been treated by anterior resection or abdominoperineal resection [54, 57]. In a study of quality of life (QoL) in patients who underwent pelvic exenteration for rectal cancer [57], a comparison was made between patients undergoing standard therapy for rectal cancer which was not locally advanced (anterior resection/abdominoperineal resection) compared to those with locally advanced disease who had a “curative” pelvic exenteration. On two separate quality- of- life scores (SF36 and FACT-C), there were broadly comparable QoL scores in both tools, with FACT-C scores of 107.3 versus 106.4 in the pelvic exenteration and low anterior resection/APE groups, respectively.

## 18. Outcome Measures

Early reports of outcomes following pelvic exenteration demonstrated poor quality of life together with significant levels of morbidity and mortality following surgery [4]; however, more recently there have been substantial improvements in outcome following this type of surgery [42, 48, 50, 58–60]. In a 2007 study by a group in Rotterdam [61], the authors published their 10-year outcomes for TPE. Over the study period a total of 23 patients underwent TPE for locally advanced primary rectal carcinoma, 19 patients (82.6%) had a negative CRM following surgery, major morbidity was seen in 6 patients (26.1%), and overall complication rate was 60.9%. Local control was 88% at 5 years, and overall 5-year survival was 52%. Univariate analysis predicted adverse outcomes for both survival and local recurrence with positive resection margins and the presence of preoperative pain. 

The authors have previously published their results of exenterative surgery from all forms of pelvic oncology [11] which demonstrated that over the initial eleven years (1992–2003) of the Swansea Pelvic Oncology group 130 patients were assessed of which 76 underwent surgery with an intent to cure. Of these 21 were converted to a palliative procedure intraoperatively due to irresectable disease (27.6%). Failure to undertake a curative resection was usually due to sacral or vascular adherence by tumour invasion. Clear potentially curative resection margins were achieved in 40 of the 55 (72.7%) patients who underwent surgery with a curative intent. Thirty- day mortality for the 76 operative cases was zero, reoperation rate for early complications was 3.9% (3 cases—bleeding, leak, and dehiscence). Ten patients required readmission for late complications including stoma problems, small bowel obstruction, wound or pelvic abscesses, and fistulation. Overall morbidity in this mixed group was 28%. Follow-up of all 130 patients assessed for potential multivisceral resection ranged from 1 to 120 months, with a median survival in patients not undergoing surgery of 12 months, and there was not statistically significant difference in survival between patients not undergoing surgery and those undergoing surgery with palliative intent and those having surgery with a positive resection margin on histological assessment. Survival in the patients with clear histological margins was significantly improved at 53% at 5 years.

More recently, the authors have published their results with regards to multivisceral resection of locally advanced primary rectal carcinoma [12]. This study reports the groups' results from multivisceral resection for purely T4 primary rectal carcinoma. Between 2000 and 2009 a total of 42 locally advanced primary rectal carcinomas underwent surgical treatment with intent to cure. Thirty- one patients were male and 11 female, age range from 41 to 83 (median 62). Twelve were upper rectal cancers, 19 midrectal, and 11 low rectal. Follow-up ranged from 2 to 102 months (median 30). Of these 31 underwent neoadjuvant therapy with the remainder not receiving pre-operative chemoradiotherapy treatment due to tumour position (10 upper rectal carcinomas) and 1 having received previous prostatic radiotherapy. Fifty-five percent had a local response to therapy. Of the surgical procedures there were 23 cystoprostatectomies, 7 en bloc bladder sparing radical prostatectomies, 10 radical hysterectomies, and 1 sacrectomy. Pelvic reconstruction in this group included 7 bilateral gracilis muscle flaps, 29 omentoplasties, 2 porcine collagen implants, and 3 pedicled musculocutaneous flaps (2 VRAM, 1 anterolateral thigh/vastus lateralis). An R0 resection being achieved in 93% with nodal disease was found in 69% and extramural vascular invasion in 33%. Thirty- day hospital mortality was zero, and overall morbidity was 49%, 21% developing pelvic collection or abscess, 10% anastomotic leak, 5% small bowel obstruction, and 2% for each of vesicoperineal fistula, spinal cord ischaemia, rectovaginal fistula, and incisional hernia. A total of 8 patients (19%) had reoperation for complications.

Local recurrence rate in this series was 7% after a median disease-free interval of 19 months. Five-year overall survival in patients with complete resection was 48% which compares to 33% for those with residual local disease and zero at 3 years for those with metastatic disease. In this study adverse outcome was associated on univariate analysis with metastatic disease, nodal metastases, and extramural vascular invasion and the development of local recurrence.

There are many other studies which report the outcome of exenterative surgery from around the world; a number of these are shown in Table 2. As can be seen morbidity is substantial in most series and varies between 27% and 83%; however, 30-day in-hospital mortality is generally low (0%–9%), which is a significant improvement on earlier published results. This is despite an increasingly elderly population with more frequent comorbidities, which is a reflection of the improvements in peri- and postoperative care facilities. Five-year survival varies between 20% and 83% being higher in the recurrent tumour series and in series requiring sacrectomy. Local recurrence varies between 7 and 85%. Features which have been associated in these studies with an adverse outcome include the presence of metastases at surgery, node-positive disease, extramural vascular invasion, positive circumferential resection margins, and poor differentiation amongst others. Consistency in analysis of prognostic factors predicting adverse outcome is poor, probably due to differences in factors analysed in each study and the low numbers of cases analysed in many of the published series.

## 19. Conclusions

Exenterative surgery for locally advanced and recurrent rectal cancers is an established technique for the small number of patients in each cancer network per year. Management needs to be tailored to the individual patient drawing on the experience of the multi-speciality pelvic oncology team. The role of preoperative planning and careful case selection cannot be underestimated. Oncological outcomes are maximised in cases in which clear resection margins are achieved and in the absence of markers of systemic disease such as node positive disease or extramural lymphovascular invasion. The authors consider that optimal outcomes can only be achieved in centres with institutional expertise in this challenging yet rewarding patient group.

## Figures and Tables

**Table 1:** Classification of anatomical location of pelvic tumour.

Moore et al. [29]	Axial (perineal location; anastomotic recurrence)
	Anterior (urological/gynaecological organ involvement)
	Posterior (sacrum/coccyx involvement)
	Lateral (pelvic sidewalls)

Yamada et al. [39]	Localized (adjacent pelvic organs or connective tissue)
	Sacral (S3–5, coccyx, or periosteum)
	Lateral (lateral pelvic wall, S1 or 2, sciatic nerve, or greater sciatic foramen)

Royal Marsden	Central
	Posterior/sacral
	Lateral
	Inferior/perineal
	Anterior
	supraperitoneal
	at peritoneum
	infraperitoneal

**Table 2:** Outcomes in pelvic exenteration surgery.

Study	*N*	Tumour type	Procedure type	Morbidity	Mortality	5-year survival	LR rate	Adverse prognostic features
Austin and Solomon [31]	36	All pelvic	All	70%	0%	69%	28%	n/a
Bakx et al. [43]	40	Rec. rectal	All	83%	6%	28%	n/a	Positive CRM, symptomatic recurrence at surgery, peripheral recurrence
Bannura et al. [44]	30	1° rectal	PPE	50%	0%	48%	30%	n/a
Derici et al. [40]	57	1° rectal	All	32%	3.5%	49%	18%	Positive CRM, node positive
Eckhauser et al. [45]	12	1° rectal	PPE, TPE	75%	8%	50%	36%	n/a
Ferenschild et al. [46]	69	All pelvic	TPE	67%	0%	38%	30%	Positive CRM
Ferenschild et al. [47]	25	1° and rec. rectal	All and sacrectomy	68%	0%	30%	66%	Node positive, positive CRM
Gannon et al. [7]	45	1° and rec. rectal	PPE, TPE	47%	0%	52%	22%	n/a
Harris et al. [12]	42	1° rectal	PPE, TPE	49%	0%	48%	7%	Metastases, node positive, EMVI
Ike et al. [48]	71	1° rectal	TPE	66%	4%	54%	28%	Age, tumour stage, node positive,
Ishiguro et al. [49]	91	1° rectal	PPE,TPE	41%	2%	52%	54%	Lateral pelvic LN positivity, raised CEA, EMVI
Larsen et al. [41]	124	1° rectal	PPE, TPE	41%	2.5%	52%	15%	Age, annular tumour, poor differentiation, pelvis side wall resection, transfusion
Law et al. [50]	24	1° and rec. rectal	All	54%	0%	44%	n/a	n/a
Maetani et al. [51]	61	Rec. rectal	All	n/a	3%	28%	41%	n/a
Melton et al. [52]	29	Rec. rectal	TPE and sacrectomy	59%	3.5%	20%	85%	Transfusion, positive CRM, bone invasion, anterior organ invasion
Moriya et al. [53]	128	1° rectal	All	27%	1%	57%	34%	BMI, node positive, inflammatory reaction
Nguyen et al. [11]	76	All pelvic	All	28%	0%	53%	n/a	n/a
Roos et al. [54]	35	1° and rec. rectal	All	69%	3%	52%	12%	Positive CRM, preoperative pain
Verschueren et al. [55]	11	1° rectal	PPE, TPE	27%	9%	68%	9%	n/a
Wiig et al. [56]	6	1° rectal	LAR/APR and prostatectomy	66%	0%	83%	n/a	n/a
Påhlman et al. [33]	28	All pelvic	All	54%	7%	n/a	n/a	n/a
Yamada et al. [5]	64	1° and rec. rectal	TPE, PPE, and sacrectomy	56%	2%	39%	n/a	n/a

APE: anterior pelvic exenteration, PPE: posterior pelvic exenteration, TPE: total pelvic exenteration, All: APE, PPE, TPE, LAR: low anterior resection, APR: abdominoperineal.
